# The Impact of Family Intactness on Family Functioning, Parental Control, and Parent–Child Relational Qualities in a Chinese Context

**DOI:** 10.3389/fped.2014.00149

**Published:** 2015-01-29

**Authors:** Daniel T. L. Shek, Qiuzhi Xie, Li Lin

**Affiliations:** ^1^Department of Applied Social Sciences, The Hong Kong Polytechnic University, Hong Kong, China; ^2^Public Policy Research Institute, The Hong Kong Polytechnic University, Hong Kong, China; ^3^Department of Social Work, East China Normal University, Shanghai, China; ^4^Kiang Wu Nursing College of Macau, Macau, China; ^5^University of Kentucky College of Medicine, Lexington, KY, USA

**Keywords:** family intactness, parenting process, family functioning, behavioral control, psychological control, parent–child relational qualities

## Abstract

The current study investigated the differences between intact and non-intact families in family processes, including systematic family functioning, parental behavioral control, parental psychological control, and parent–child relational qualities. The participants were 3,328 Secondary One students, with a mean age of 12.59 years, recruited from 28 secondary schools in Hong Kong. Four validated scales were used to assess family processes. Results showed that adolescents in non-intact families perceived relatively poorer family functioning, lower level of paternal and maternal behavioral control, lower level of paternal psychological control, and poorer parent–child relational qualities than did adolescents in intact families. This generally indicated that family processes were poorer in non-intact families, compared with those in intact families. The theoretical and practical implications of the findings were discussed.

## Introduction

A large amount of research has shown that relative to children living in intact families (i.e., the families that include two married biological parents), children living in non-intact families (i.e., the families that are not consisted of two married biological parents) are more likely to exhibit negative psychological and educational outcomes such as poorer academic performance, more risk behaviors, and declined subjective well-being (1–5). It is to be noted here that two-parent adoptive families should not be considered as intact families, because these adoptive families tend to differ from intact non-adoptive families in family functioning and the incidence of children’s psychological risks (6). However, in Hong Kong adoptive families are rare and non-intact families are primarily concerned with divorced and conjugally separated families. A number of theories such as family ecological theories (7) and social control theories (8) suggest that parental marital disruption adversely affects child development through deteriorated family environment and parenting processes. Shek and Leung (5) summarized three main points in these theories that explain how marital disruption influences child development and parenting processes. First, marital disruption may dampen parents’ well-being, which in turn adversely affects their parenting, such as parental discipline and monitoring. Second, family disruption may lead to family financial difficulty. In this situation, if the single-parent has to work for a living, parental supervision over the child may further be reduced. On the other hand, if the parent receives welfare, economic disadvantage may also adversely affect parenting processes. Third, marital disruption brings parents more stresses, which may lead to adjustment difficulties for parents and deteriorated parenting processes.

Parenting processes are important for adolescent development and could be represented by family functioning, parental behavioral control, parental psychological control, and parent–child relational qualities. Family functioning denotes to what extent family members are bonded emotionally, communicate effectively, and respond to problems collaboratively (9). Parental behavioral control refers to the regulations and constraints that parents exert on their children and parents’ awareness of their children’s behaviors (10). Parental behavioral control can be divided into five dimensions: parental knowledge about child, parental expectations on child’s behaviors, parental monitoring, discipline, and demandingness (11–13). Parental psychological control refers to parents’ attempts to exert control over their children through interfering or manipulating their children’s feelings and thoughts (14). Research showed that higher levels of family functioning and behavioral control were associated with positive adolescent educational and psychological outcomes; whereas higher level of psychological control was related to negative outcomes (15, 16).

A number of studies demonstrated that family functioning was better in intact two-parent families than in single-parent families (17, 18); whereas other studies did not show such tendency (19, 20). Freistadt and Strohschein (21) as well as Brown and Manning (22) further looked into family functioning in cohabiting step-families in addition to single-parent families. They found that family functioning was poorer in single-parent and cohabiting parents’ families relative to families composed of two married biological parents.

The findings on the effect of family structure on parental behavioral control and psychological control are also inconclusive. For example, Florsheim et al. (23) found that the level of parental monitoring was lower in single-mother families than in two-parent families. Pettit et al.’s (24) study showed that mothers in two-parent families reported stronger parental monitoring than did single mothers, but this tendency was not demonstrated in adolescent-reported monitoring. However, some researchers reported that family structure was associated with neither adolescent-reported nor mother-reported parental monitoring (10, 25). Shek (13, 26) explored parental behavioral control in five dimensions and revealed that parental knowledge, expectation, monitoring, discipline, and demandingness were weaker in non-intact families than in intact families; and that this tendency was more obvious in fathers’ parenting than in mothers’ parenting. Shek (26) further reported that the level of parents’ behavioral control was stronger in single-parent non-intact families than in re-married non-intact families. Regarding parental psychological control, several studies did not show the relationship between family structure and parental psychological control (10, 24, 25). However, Shek (13, 26) reported that maternal psychological control was stronger in non-intact families than in intact families, but Shek’s (26) study did not show the difference in parental psychological control between single-parent families and re-married families.

A number of studies showed that parent–child relationships were generally better in intact families than in non-intact families (13, 26–28), and the negative influence of divorce on parent–child relationships can last for a long period of time (29, 30). Additionally, Kalmijn’s (30) research concluded that although children’s relationships with fathers and with mothers are both affected by divorce, divorce is likely to increase inequality in the relations with parents. In a divorced family, a child may have a poor relation with one parent but keep a good relation with the other parent. Kalmijn (30) interpreted that the inequality in the relations may be out of loyalty conflicts and compensation effects. In experiencing conflicts of loyalty, children may interact more with one parent and disengage from the other parent. Alternatively, one parent may invest more in a child when the relation between the child and the other parent deteriorates.

Fathers’ and mothers’ parenting in divorced families are likely to be different. Residential mothers are inclined to be more openly communicating with their children and more involved in monitoring and knowing their children, whereas residential fathers tend to have less custody stress and may have fewer problems with parental discipline and control (31). Furthermore, divorce tends to undermine opposite-sex relationships more than same-sex relationships. However, O’Connor et al.’s (32) study showed that father–son relationship was more conflicted than father–daughter relationship in non-intact families. In addition, non-residential mothers tend to have more frequent contact with children than do non-residential fathers.

To summarize, the existing findings on the effect of family structure on family functioning and parental control are quite inconsistent. The inconsistency could be explained by the following reasons. First, these studies were conducted in different cultural contexts that may influence the associations between family structure and parenting processes. While most studies conducted in the Western context suggested that children in different types of families did not report different level of parental control (23, 24). Shek’s (13, 26) studies conducted in a Chinese context indicated that children in non-intact families reported weaker parental behavioral control and stronger maternal psychological control. Such difference may be due to the different cultural perspectives on marital disruption. In the Chinese cultural context, divorce is regarded as more negative and family intactness is considered to be more important than in the Western cultural context. This may result in different psychological states of divorced parents, which in turn affect parenting. Second, scholars inconsistently categorized cohabiting step-families. Some studies combined them with single-parent families, whereas others included them in two-parent families. The inconsistent classification of family types may lead to contradictory findings (21). Third, some studies did not examine paternal control and maternal control separately (23, 24). If paternal control and maternal control over their children are apparently different, children may find it difficult to answer the questions regarding parental control and parent–child relationships. As a result, some children may answer these questions mainly according to maternal parenting, whereas others mainly according to paternal parenting.

The current study attempted to examine the effect of family intactness on family functioning, parental control, and parent–child relational qualities among junior secondary school students in Hong Kong. It also addressed the above-mentioned limitations in the existing studies. First, this study was conducted in a Chinese cultural context. As most of these studies were conducted in the Western cultural context and there is a dearth of studies conducted in the Chinese cultural context (13, 26), our study supplemented the insufficient literature and contributed to the understanding of the effect of family intactness on parenting processes in the Chinese cultural context. Second, our study included the cohabiting step-families in the family type of non-intact families, given the evidence that parenting processes in cohabiting step-families tended to be poorer than those in families composed of two biological parents (21, 22). Third, our study investigated fathers’ and mothers’ parenting processes (i.e., parental behavioral control, psychological control, and parent–child relational qualities) separately.

Furthermore, as most of the existing studies examined parental behavioral control merely in the dimension of parental monitoring, studies assessing more dimensions of parental behavioral control are needed (13). Our study examined parental behavioral control in three important dimensions: parental knowledge, expectation, and monitoring. Additionally, Freistadt and Strohschein (21) pointed out that many studies had a methodological problem that they were based on small and non-representative samples. For instance, some studies involved fewer than 100 adolescent participants (10, 19), and some others involved the participants from merely one specific district of a city (17, 18). To address this problem, our study used a large sample of adolescents who were randomly selected from dozens of secondary schools across different districts of Hong Kong.

Based on the existing findings and the anticipation that parenting processes in non-intact families tend to be poorer than those in intact families in the Chinese cultural context, we made the following hypotheses:

Hypothesis 1: Family functioning would be better in intact families than in non-intact families.
Hypothesis 2: The level of maternal and paternal behavioral control would be higher in intact families than in non-intact families.
Hypothesis 3: The level of maternal and paternal psychological control would be lower in intact families than in non-intact families.
Hypothesis 4: Mother–child and father–child relationships would be better in intact families than in non-intact families.

## Materials and Methods

### Participants

This study is part of the extension phase of the Project Positive Adolescent Training through Holistic Social Programs (P.A.T.H.S.), which is a pioneering positive youth development program aiming to promote holistic development among Hong Kong youth. The participants were recruited from 28 secondary schools, which were randomly selected from all the Government and aided secondary schools in Hong Kong. We had collected multi-wave data for the Project P.A.T.H.S., and the Wave 1 data were used in the current study.

The participants of this study were 3,328 Secondary One students with a mean age of 12.59 years (SD = 0.74). Among these participants, 1,719 students were males, 1,572 were females, and 37 students did not report gender. Ethical approval from The Hong Kong Polytechnic University as well as parents’ and schools’ written consent was obtained prior to the data collection. We also obtained students’ written consent before the data collection and emphasized anonymity and confidentiality.

### Instruments

We used the scales of family functioning, parental behavioral control, parental psychological control, and parent–child relational qualities. Because many questionnaires were used in the data collection for the Project P.A.T.H.S to obtain various information about adolescents, shortened versions of the scales were used in order to reduce administration time.

#### Assessment of household demographics

Parental marital status was assessed by a five-option item. The five options are: (1) divorced but not re-married, (2) separated but not re-married, (3) married (first marriage), (4) married (second or above marriage), and (5) others. Participants were also asked to indicate whether they lived with father and whether they lived with mother. In addition, the information regarding the number of participants’ household members and father’s and mother’s education levels was collected.

#### Chinese family assessment instrument

The self-reported Chinese family assessment instrument (CFAI) measures family functioning in the Chinese cultural context (33). It was developed among Hong Kong adolescents based on the McMaster model of family functioning (34), the review of the existing family functioning assessment, and the research findings on Chinese people’s perception of happy family characteristics (35). Nine items in three subscales (i.e., Mutuality, Conflicts, and Communication) in the original instrument were used in this study. Each subscale contains three items, and a 5-point Likert scale is used for scoring (1 indicates *very dissimilar to my family situation* and 5 indicates *very similar to my family situation*). Previous studies reported that this instrument has good reliability and validity among the youth in Hong Kong and Mainland China (36–38). In this study, Cronbach’s alpha coefficients were 0.87, 0.76, and 0.81, respectively for the subscales of Mutuality, Conflicts, and Communication.

#### Assessment of parental behavioral control

This self-reported scale was developed by Shek (39) for Hong Kong adolescents. A shortened seven-item version of this scale was used in this study, and it contains three subscales: parental knowledge (two items), expectation (two items), and monitoring (three items). The items were scored from 1 (*strongly disagree*) to 4 (*strongly agree*). Higher scores indicate stronger parental behavioral control. Good psychometric properties of this questionnaire have been reported (26, 39). As the Spearman–Brown coefficient is less likely to underestimate true reliability or biased for two-item scales than Cronbach’s alpha (40), we reported the Spearman–Brown coefficients instead of the commonly used Cronbach’s alpha for internal consistency reliability of the subscales measuring parental knowledge and parental expectation. The internal consistency reliability coefficient ranged from 0.68 to 0.87 for the subscales in the current study.

#### Assessment of parental psychological control

The abridged four-item version of the Chinese Psychological Control Scale (39) was used to measure parental psychological control. A four-point Likert scale from 1 (*strongly disagree*) to 4 (*strongly agree*) was used, and a higher score represents higher level of parents’ psychological control. Previous studies reported good psychometric properties of this scale (26, 39). Cronbach’s alpha coefficients were 0.80 for paternal psychological control and 0.85 for maternal psychological control in this study.

#### Assessment of parent–child relational qualities

This scale was developed by Shek (39). A shortened version was used in this study, and it is composed of two subscales: satisfaction with parental control (three items) and parent–child relationships (three items). Each item is scored from 1 (*strongly disagree*) to 4 (*strongly agree*). Higher scores indicate better parent–child relational qualities. The psychometric properties of this assessment were reported to be good in previous studies (13, 26, 39). Cronbach’s alpha coefficients ranged from 0.85 to 0.88 for the subscales in this study.

## Results

The current sample included 2,616 adolescents from intact families and 535 adolescents from non-intact families, which included parents who were divorced (*n* = 194), separated (*n* = 221), re-married (*n* = 35), or engaged in other non-husband-wife relationships (*n* = 79). The rest of six adolescents from non-intact families did not report their parents’ marital status. Moreover, we could not identify family intactness of the other 177 adolescents due to inadequate family information. Although they reported living with both parents, they either did not report parents’ marital status (*n* = 26) or reported that their parents were married but not in the first marriage (*n* = 151). In this case, they might be born in the second marriage of their parents, indicating they were in intact families, or born in the first marriage of one parent, indicating they were in non-intact step-families.

Table 1 shows the descriptive statistics and the results of three MANCOVA tests on the effects of family intactness on family functioning, paternal parenting, and maternal parenting. In the MANCOVA tests, family intactness was the independent variable and the variables related to family functioning, paternal parenting, and maternal parenting were the dependent variables. Besides, adolescents’ age, number of household members, and parental education levels were selected as the covariates in the MANCOVA tests because preliminary data analyses show that only these demographics were significantly correlated with some of the dependent variables. Intact and non-intact families differed in all of the four domains of parenting (see Table 1).

**Table 1:** **Effects of family intactness on family functioning, parental control, and parent–child relational qualities**.

Variables	Mean	SD	Reliability	Intact		Non-intact		Mean square	*F*	Effect size
				Mean	SD	Mean	SD	
Family functioning									Omnibus F 39.44**	0.041
Mutuality	3.90	0.89	0.87	3.98	0.86	3.51	0.97	86.38	112.16**	0.038
Conflicts	2.17	0.92	0.76	2.11	0.90	2.48	0.93	54.76	66.37**	0.023
Communications	3.51	1.01	0.81	3.59	0.98	3.13	1.05	79.80	80.81**	0.028
Paternal parenting									Omnibus F 25.80**	0.057
Paternal knowledge	2.50	0.78	0.83	2.58	0.74	2.13	0.84	69.23	120.35**	0.045
Paternal expectation	2.74	0.74	0.68	2.79	0.71	2.48	0.87	31.84	58.67**	0.022
Paternal monitoring	2.49	0.80	0.86	2.54	0.77	2.22	0.88	33.70	53.90**	0.021
Paternal psychological control	2.26	0.73	0.80	2.28	0.71	2.18	0.80	3.17	5.98*	0.002
Satisfaction with paternal control	2.93	0.73	0.88	2.99	0.68	2.63	0.86	45.70	89.16**	0.034
Father–child relationship	2.67	0.82	0.85	2.74	0.78	2.30	0.90	66.26	103.09**	0.039
Maternal parenting									Omnibus F 10.69**	0.022
Maternal knowledge	3.05	0.75	0.86	3.10	0.71	2.82	0.87	29.98	54.78**	0.019
Maternal expectation	3.03	0.68	0.68	3.05	0.67	2.88	0.75	11.42	24.55**	0.009
Maternal monitoring	3.00	0.74	0.87	3.04	0.71	2.79	0.84	23.93	44.71**	0.016
Maternal psychological control	2.34	0.79	0.85	2.33	0.78	2.38	0.83	1.01	1.64	0.001
Satisfaction with maternal control	3.10	0.68	0.88	3.12	0.67	2.98	0.75	8.23	17.75**	0.006
Mother–child relationship	2.99	0.80	0.87	3.03	0.77	2.80	0.88	20.10	32.16**	0.011

*Intact, intact families; non-intact, non-intact families; reliability, internal consistency reliability coefficient. The Spearman–Brown coefficients were calculated for the reliability of the subscales of Paternal knowledge, Paternal expectation, Maternal knowledge, and Maternal expectation; whereas, Cronbach’s alpha coefficients were calculated for the reliability of the other subscales*.

*p < 0.05, **p < 0.001.

Regarding family functioning, adolescents in intact families scored higher on perceived family mutuality and communications, meanwhile, they also scored lower on perceived family conflicts than did adolescents in non-intact families. These results support our Hypothesis 1. Compared with adolescents in non-intact families, adolescents in intact families scored higher on all the three dimensions of paternal and maternal behavioral control (i.e., parental knowledge, expectations, and monitoring). These results support our Hypothesis 2. In addition, adolescents in intact families scored higher on paternal psychological control than did adolescents in non-intact families, and this was contrary to our Hypothesis 3. Although adolescents in intact families scored lower on maternal psychological control than did adolescents in non-intact families, the difference did not reach significant level. Finally, the results showed that adolescents in intact families were more satisfied with the control of both parents and they also perceived better father–child and mother–child relationships compared with adolescents in non-intact families. These results support our Hypothesis 4.

## Discussion

This study examined the differences between intact and non-intact families in family functioning, parental behavioral control, parental psychological control, and parent–child relational qualities. The present study has several strengths. First, this study supplements the insufficient literature about the associations between family structure and parenting processes conducted in the Chinese cultural context. Second, this study investigated parental behavioral control in three dimensions. As many studies investigated parental behavioral control merely in the dimension of parental monitoring, our study was able to examine the associations between family structure and parental behavioral control in a more holistic manner. Third, our study investigated fathers’ and mothers’ control as well as father–child and mother–child relationships, respectively. In so doing, adolescents did not find it difficult to respond to the relevant measurement items when their fathers’ and mothers’ parenting were different. Also, the results obtained from this study could enable us to compare fathers’ and mothers’ parenting processes in different family types. Fourth, the sample size in this study is large and representative, because we selected participants randomly from all the local secondary schools.

Our study has several important findings. First, to our best knowledge, this is the first study investigating the effect of family structure on perceived family functioning in a Chinese context. The results show that intact families had more mutuality and communication as well as less conflict than did non-intact families. This indicates that compared with intact families, non-intact families in Hong Kong have poorer family functioning.

Second, the levels of both paternal and maternal behavioral control in non-intact families were lower than those in intact families. These results are consistent with Shek’s (13, 26) earlier findings obtained in Hong Kong and indicate that non-intact families in Hong Kong are likely to be more limited in the ability to exert regulations on children than are intact families.

Third, among the parenting processes measured in this study, the difference in psychological control was smallest between intact and non-intact families. Compared with those in non-intact families, adolescents in intact families scored higher on paternal psychological control; however, this tendency was not shown to be significant for maternal psychological control in this study. These findings imply that marital disruption may have different effects on paternal and maternal psychological control. It may be due to the fact that among the adolescents in non-intact families in our sample, many (*n* = 365) of them only lived with their mothers and the absence of fathers in family life reduced the perceived level of paternal psychological control.

Finally, regarding parent–child relational qualities, the current study shows that compared with adolescents in non-intact families, adolescents in intact families were more satisfied with paternal and maternal control and they also had better relationships with both parents. These results are consistent with earlier findings (13, 26, 32) and corroborate that parent–child relational qualities are better in intact families than in non-intact families.

To summarize, this study indicates that intact families generally have better parenting processes than do non-intact families. Such findings can be explained by the following possible reasons. Most obviously, in many non-intact families, only a single-parent takes care of child and the absence of a second parent possibly results in limited parenting capacity. Second, non-intact families are not widely accepted in the Chinese society, and parents in non-intact families may be subject to unfavorable reputation, which in turn adversely affects their affective interactions with children (13). Third, Freistadt and Strohschein’s (21) study suggested that single-parent families and cohabiting step-families had lower extra-familial social capital. As a result, parents in non-intact families may invest more time and effort to maintain extra-familial social networks at the expense of the involvement in their own families (21). Furthermore, the existing marital theories suggest that family members in non-intact families are more likely to encounter financial difficulty and psychological stresses, which potentially lead to declined parenting capacity (5).

The current findings support some family theories, such as the family system theories (41), explaining that parenting may be impaired in non-intact families. By demonstrating the differences between intact and non-intact families in a wide range of parenting processes, the present findings are conducive in building theoretical models to describe more specifically parenting differences between intact and non-intact families (13).

This study also has practical implications. The findings of our study provide a useful pointer for professional practitioners by informing them the details of the difference between intact families and non-intact families in specific parenting. Besides, our study suggests that parenting is comparatively poorer in non-intact families than in intact families, thus implying that family life education and parenting programs are necessary to be designed to help parents who have experienced marital disruption to be aware of the possible changes in parenting as well as to improve their parenting skills and their relationships with children.

The study has several limitations. First, because we used abridged versions of the parenting instruments, some subscales of the measurements used in this study merely contain two or three items. Although good internal consistency reliability was reported for each scale, psychometric properties, especially content and construct validities, of the abridged versions were likely to be poorer than those of the full versions, which were demonstrated to be good (42). Therefore, future studies that assess parenting processes should use the full version of the scales. Second, the parenting processes examined in this study were based on adolescents’ reports. Thus, the findings may not precisely indicate the real parenting processes but mainly represent adolescents’ perceived parenting processes. Third, the study was conducted in Hong Kong. To have a more insightful understanding of the differences between intact and non-intact families in parenting processes in the Chinese cultural context, more such studies should be conducted in other areas of China. Fourth, we only divided family types into intact and non-intact families and did not further classify non-intact families into sub-types such as single-parent families and step-families due to the fact that the number of step-families (*n* = 35) in our study was too small as opposed to single-parent families (*n* = 415). Hence, our study has the limitation in providing more informative findings on the relationships between family structure and parenting processes. Last, as we only used single-wave data, our study cannot indicate causal relationships between family intactness and parenting processes as well as the long-term effect of family structure on parenting processes. Longitudinal studies that use the information of family structure at an earlier time to predict parenting processes at later times are needed.

## Conflict of Interest Statement

The Associate Editor Hatim A. Omar and the Review Editors Leslie A. Appiah, Alissa Briggs, and Marlene Belew Huff declare that, despite being affiliated with the same institution as Daniel Tan Lei Shek, the review process was handled objectively. The authors declare that the research was conducted in the absence of any commercial or financial relationships that could be construed as a potential conflict of interest.
